# Diseases of the Liver: With and without Jaundice

**Published:** 1883-07

**Authors:** 


### Notes on Books.

*Diseases of the Liver: with and without Jaundice.* By Dr. George Harley, F.R.S. Pp. 1200. London: J. & A. Churchill, 1883.

We confess to have read this work with very mingled feelings—not without interest certainly—but with disappointment and some regret.

Dr. Harley is a man of pronounced opinions. He has a very strong belief in himself, and he lays down the law with an amount of dogmatism from which there is no escape, but which seems hardly wise in a physician of his experience.

It would be impossible for us in a brief review to give a comprehensive analysis of Dr. Harley's book. It is a large and pretentious volume, and at first sight the reader is rather appalled at the prospect of going through it from cover to cover, but the type is remarkably large, and the amount of matter in each page, is consequently not great.

Dr. Harley distinctly states that his book is specially intended for his medical brethren. Were it not for that statement we should have been disposed to think it was principally directed to catch the popular ear.

The author uses strong language. He says "that even in highly educated Germany scientific medicine is still in its infancy, while in England it may be said to be still in its long clothes." He fires a shot at Clinical Teachers in the Metropolitan Schools, who still lecture to their pupils on what they please to term "Functional Derangements of the Liver"—which he evidently does not believe in—and taunts them with the assertion that while they are pursuing what he terms a "pernicious system," they would repudiate with scorn the idea of prescribing for a palpitation of the heart, a bronchial expectoration or purulent urine under the title of functional disease of the Heart, Lungs, or Kidneys.

We would only remark that an eminent and distinguished Physician and Clinical Teacher, not long deceased, whose experience of Liver Disease, both in India and at home, must at least have been equal to Dr. Harley's, taught that there *was* such a thing as Functional Derangement of the Liver, and taught it effectually.

On the vexed question of Jaundice from Suppression Dr. Harley is intolerant of opposition, and goes so far as to assert without mercy that he who doubts in its existence "cannot have the slightest pretence to the possession of a clear judgment," and that the theory "must for ever cease to be regarded either as a clinical or pathological myth." We venture to call this statement in question, in so far at all events that there are very strong arguments on the other side. There is absolutely no reliable evidence to show that bile exists already formed in the blood, not even in that of the portal vein. Cases are on record where almost the whole liver has been destroyed, leaving nothing but the capsule—by suppuration—without Jaundice, and the experiments of Mueller, Kunde and Moleschott are entirely opposed to the suppression theory of Jaundice.

The Jaundice from suppression which Dr. Harley believes in is a very different thing from the Hæmatogenous Cholæmia, the result of disintegration of blood corpuscles manifested under such conditions as Pyæmia, Yellow Fever, Acute Yellow Atrophy and Snake Bite.

It is a pity that a work, which certainly possesses some merits, should be defaced with faults quite unworthy of the author's standing and position as a physician of experience. What for example can be more full of clap-trap than his chapter on Dry Champagne, upon which beverage he vents his wrath? Those who drink it, he says, "live in a fool's paradise and turn their stomachs into pickle jars." But he takes care to tell us that his own champagne is A 1, "as his friends very well know." Would that we could number ourselves among these happy mortals! But why deface a work on medical science with stuff of this kind?

The chapter on Contagious Jaundice is a remarkable one. This is to be, or ought to be, in Dr. Harley's opinion, the new name for that terrible disease, Yellow Fever; for he says "it is nothing more than an ordinary form of Jaundice from hepatic disease of a more than usually severe type."

That Yellow Fever and Acute Yellow Atrophy of the Liver have some features in common we do not deny. But that Yellow Fever, the most pestilential and destructive form of contagious fever known, is nothing more than an aggravated form of Jaundice is a statement which we cannot endorse. Jaundice is but a symptom of this Fever, one out of many of general and specific blood poisoning. The amount of discoloration of the skin bears no proportion to the gravity of the general symptoms of Jaundice *per se* usually present, for the fæces still retain their bilious colour, and it has never been possible to demonstrate the presence of biliary acids in the blood of Yellow Fever patients. Surely the most tenable hypothesis of this disease is that a specific poison germ has been introduced into the circulation which produces solution and destruction of blood corpuscles, and a conversion of their hæmatine into bile pigment—in other words, Hæmatogenous Cholæmia.

We have no space to pursue the examination of Dr. Harley's work farther. We should be sorry to give an unfavourable impression of every part of it. Some chapters will repay the reader, especially that on Abscess of the Liver. But the value of the book is injured by conjectural theories advanced without sufficient scientific proof and by dogmatic assertions which offend the taste, while they do not convince the judgment.

Hence we fear that Dr. Harley's work will not add much to our knowledge of the particular subject or conduce greatly to his own reputation.

